# The Phytochemical, Quercetin, Attenuates Nociceptive and Pathological Pain: Neurophysiological Mechanisms and Therapeutic Potential

**DOI:** 10.3390/molecules29163957

**Published:** 2024-08-21

**Authors:** Mamoru Takeda, Yukito Sashide, Ryou Toyota, Haruka Ito

**Affiliations:** Laboratory of Food and Physiological Sciences, Department of Life and Food Sciences, School of Life and Environmental Sciences, Azabu University, 1-17-71, Fuchinobe, Chuo-ku, Sagamihara 252-5201, Kanagawa, Japan; me2401@azabu-u.ac.jp (Y.S.); zephyranthes27@gmail.com (R.T.); ito666yuu@gamil.com (H.I.)

**Keywords:** quercetin, complementary alternative medicine, nociception, inflammation, electrophysiology, voltage-gated ion channel, acid-sensing ion channel, local anesthesia, cyclooxygenase-2, non-steroidal anti-inflammatory drugs

## Abstract

Although phytochemicals are plant-derived toxins that are primarily produced as a form of defense against insects or microbes, several lines of study have demonstrated that the phytochemical, quercetin, has several beneficial biological actions for human health, including antioxidant and inflammatory effects without side effects. Quercetin is a flavonoid that is widely found in fruits and vegetables. Since recent studies have demonstrated that quercetin can modulate neuronal excitability in the nervous system, including nociceptive sensory transmission via mechanoreceptors and voltage-gated ion channels, and inhibit the cyclooxygenase-2-cascade, it is possible that quercetin could be a complementary alternative medicine candidate; specifically, a therapeutic agent against nociceptive and pathological pain. The focus of this review is to elucidate the neurophysiological mechanisms underlying the modulatory effects of quercetin on nociceptive neuronal activity under nociceptive and pathological conditions, without inducing side effects. Based on the results of our previous research on trigeminal pain, we have confirmed in vivo that the phytochemical, quercetin, demonstrates *(i)* a local anesthetic effect on nociceptive pain, *(ii)* a local anesthetic effect on pain related to acute inflammation, and *(iii)* an anti-inflammatory effect on chronic pain. In addition, we discuss the contribution of quercetin to the relief of nociceptive and inflammatory pain and its potential clinical application.

## 1. Introduction

Complementary alternative medicine (CAM) therapies, such as herbal medicines and acupuncture, are often used in pain management, especially after a failure of conventional Western medicine or when adverse side effects are a concern [1,2,3,4]. CAM is defined by current Western medicine as a medical system that has not been scientifically tested and clinically applied. Phytochemicals are plant-derived toxins, primarily produced by plants as a form of defense against insects or microbes. Nonetheless, certain chemical compounds found in plants, such as the phytochemicals present in foods, possess diverse advantageous biological properties, including anti-cancer, antioxidant, anti-arteriosclerotic, and blood pressure-lowering effects, for human health [5,6]. 

Currently, local anesthetics and anti-inflammatory analgesics used pharmacologically in medicine for pain relief are known to have side effects in addition to their main effects. For example, before dental operations, such as tooth extraction, local anesthetic agents are often applied to the patient. However, if blood levels rise too high during the dental operation, adverse side effects on the central nervous system or cardiovascular system may occur [7]. Also, it is known that the presence of inflammation decreases local anesthetic efficacy, especially with dental anesthetics [8]. Although non-steroidal anti-inflammatory drugs (NSAIDs) are potent and effective inhibitors of cyclooxygenase-2 (Cox-2) for analgesia, unfortunately, pharmacological treatment of pain using NSAIDs causes several side effects, including stomach ulcers and heart attacks [9]. Therefore, phytochemicals are expected to be effective candidates that can contribute to CAM by providing pain relief to inflamed areas without side effects. 

One of the phytochemicals, quercetin, is a prevalent flavonoid found in fruits and vegetables and present in the everyday human diet [10]. Quercetin possesses a range of biological activities, including, antioxidant, anti-inflammatory, and cardioprotective properties [6,11,12]. Recent studies have demonstrated that quercetin can modulate neuronal excitability in the nervous system, including nociceptive sensory transmission via acid-sensing ion channels (ASICs), voltage-gated sodium (Nav), potassium (Kv), and calcium (Cav) channels, and inhibit the enzyme of production of prostaglandin E2 (PGE_2_) and the Cox-2-cascade. Thus, it is possible that quercetin could be a CAM candidate; specifically, a therapeutic agent against nociceptive and/or pathological pain [13,14,15,16,17,18,19,20]. Following promising in vitro experimental findings, we recently studied the pain-relieving effects of quercetin in animal experiments in our laboratory using a neurophysiological approach and have published studies demonstrating that the phytochemical, quercetin, has (i) a local anesthetic effect on nociceptive pain, (ii) a local anesthetic effect on pain related to acute inflammation, and (iii) an anti-inflammatory effect on chronic pain [18,19,20]. In this review, we discuss the contribution of the phytochemical, quercetin, to the relief of nociceptive and pathological pain and its potential clinical application on the basis of our recent in vivo studies.

## 2. Nociceptive and Pathological Pain

Pain, which typically serves as a warning signal, is referred to as physiological pain or nociceptive pain. Conversely, there is pain that no longer functions as a “biological warning signal.” This is referred to as “pathological pain”, in which alterations occur in the neurons that make up the pain transmission system, leading to persistent signal activation that causes pain in the body, greatly affecting quality of life, even after substantial tissue damage has healed [21]. Pathological pain includes inflammatory or neuropathic pain. Inflammatory pain results from the activation of nociceptors by inflammatory substances, such as PGE_2_, at the location of tissue injury, such as burns and joint pain [22]. Increased sensitivity to painful stimuli is known as hyperalgesia, whereas allodynia is the experience of pain from stimuli that are not usually painful [23]. The consensus is that these pathological pains stem from changes in the behavior of sensory neurons in somatic sensory pathways as a result of inflammation or damage to peripheral tissues [22]. Typically, peripheral sensitization, characterized by the heightened sensitivity of peripheral nerve endings and communication via chemical substances between neurons and glial cells in sensory ganglia as a result of tissue inflammation or injury, is understood to initiate central sensitization, leading to conditions like hyperalgesia [23,24,25,26].

## 3. Overview of the Trigeminal Pain Pathway

The transmission of pain signals is commonly categorized into two pathways in the trigeminal nervous system: the lateral system and the medial system [23]. Based on a range of experimental findings so far, it is believed that the lateral pain transmission pathway transmits details related to “pain intensity and location” that are experienced in the peripheral receptive field, whereas the medial pain transmission pathway conveys information about the “emotional component” of pain, such as whether it is perceived as “pleasant or unpleasant”, to a higher brain center [27]. 

An initial sensory input, such as pain, from the orofacial area (including the tongue, tooth pulp, periodontal ligament, and temporomandibular joint), which is innervated by the trigeminal nerve, is carried by trigeminal ganglion (TG) neurons to the trigeminal spinal nucleus caudalis (SpVc)/upper cervical dorsal horn (C1–C2) in the brainstem [28,29]. There are known to be two classes of neurons that respond to noxious stimuli at this specific site. Wide dynamic range (WDR) neurons are thought to relay the perception of “pain levels” from the peripheral receptive field to the central nervous system by increasing the frequency of impulse discharge in response to the intensity of stimulation in both non-nociceptive and nociceptive neurons [23,28]. These neurons may be implicated in the development of hyperalgesia following tissue damage and inflammation [23,28]. The second type of neuron, the nociceptive-specific neuron, reacts exclusively to noxious stimuli in its receptive fields, potentially transmitting location-related information to higher brain regions. Conversely, WDR neurons are responsive to both painful and non-painful stimuli [23,28]. The nociceptive information from SpVc/C1-2 contributes to the coordinated processing of the “discriminative aspects” of pain that are transmitted to the primary somatosensory cortex and the secondary somatosensory cortex via the medial ventral thalamic nucleus [30]. In contrast, nociceptive data coming from the medial thalamic nucleus are sent through the parabrachial nucleus to the limbic amygdala, insular cortex, and anterior cingulate cortex, participating in the comprehensive interpretation of the “affective component” of pain [23,31].

## 4. Peripheral and Central Transmission Mechanism of Nociceptive Pain

Primary sensory nerve fibers, including TG neurons, Aδ-fibers (the thinnest myelinated fibers with slow conduction velocities), and unmyelinated C-fibers, are implicated in the transmission of pain [23,28,29]. Aδ-fibers are responsible for carrying well-targeted, intense, stabbing pain signals rapidly, whereas C-fibers are responsible for carrying prolonged, muted pain signals that are hard to pinpoint [23,32]. TG neurons possess the morphology of pseudo bipolar cells, with the axon’s central end forming chemical synaptic connections with secondary neurons, while the peripheral end serves as a free nerve terminal and is nociceptive [23,28,29]. The nociceptors act as transducers of energy, converting external noxious stimuli (thermal, cold, mechanical, chemical energy) into electrical signals [32]. 

Sensory information in primary afferent fibers undergoes a general process involving four key steps: (i) transduction, where external stimuli are converted at the peripheral terminal; (ii) generation and initiation of action potentials; (iii) propagation through neurons that transmit action potentials; and (iv) transmission, where the central terminal plays a role in forming the presynaptic element of the first synapse in the sensory pathway of the central nervous system [28,33]. When a painful mechanical stimulus is applied to the peripheral receptive field of the skin, candidate nociceptive channels, such as transient receptor potential ankyrin 1 (TRPA1) and ASIC, are stimulated. This stimulation triggers the generation of a depolarizing potential (generator potential) as cations flow through ion channels into the cell [8,31,34]. The generator potential is a local potential that is non-conductive and acts as an analog signal with a graded amplitude based on the stimulus intensity, while the action potential serves as a conducting digital signal following the all-or-none principle. Consequently, generator potentials originating in nociceptors of primary sensory neurons are named “trigger potentials” due to their role in initiating action potentials. Noxious stimuli applied to free nerve endings can evoke potentials that exceed the threshold for generating action potentials. This initiates a depolarization phase by activating Nav ion channels on the cell membrane, causing an influx of sodium ions. The subsequent repolarization phase involves the outflow of potassium ions through Kv channels [28,35]. Both tetrodotoxin (TTX)-sensitive (S) and TTX-resistant (R) Nav channels are present in nociceptive neurons. TTX-S Nav channels and TTX-R Nav channels are found in Aδ neurons, whereas C-neurons predominantly contain TTX-R Nav channels [36].

As the intensity of the noxious stimulus applied to the receptive field escalates, so does the amplitude of the generator potential, causing a rise in the firing frequency of the ensuing action potentials [23,28]. Action potentials originating at the free nerve terminals (located at the peripheral ends of axons) travel to the central end of axons via Nav and Kv channels that are located throughout the axons. Once the action potential reaches the central end of the nerve terminal, the Cav channels at this location open, causing the nerve terminal to depolarize and permit the entry of Ca^2+^ ions. When the intracellular concentration of Ca^2+^ rises, it prompts the discharge of excitatory neurotransmitters, such as glutamate, from the presynaptic neuron into the synaptic space, allowing cations to flow into the cell by activating ionotropic glutamate receptors on the secondary sensory neurons. When glutamate receptors are activated, causing cations to flow into the cell, an excitatory postsynaptic potential (EPSP) is produced. Once this EPSP reaches a specific membrane potential threshold, an action potential is initiated. It is believed that the magnitude of the EPSP rises in proportion to the quantity of transmitter released, and the heightened rate of firing is interpreted by the central nervous system’s upper regions as data regarding the intensity of pain [23,28].

## 5. Local Anesthetic Effect of Quercetin on Nociceptive Pain

In previous studies, the effect of quercetin on excitable tissues under in vitro and in vivo conditions is summarized in Table 1. Previous in vitro experiments have shown that quercetin can influence Nav, Kv, and Cav channels in excitable tissues [13,15]. Wallace et al. [13] demonstrated an interesting observation in rat cardiac myocytes using a whole-cell patch clamp technique, indicating that quercetin, catechin, and resveratrol, found in red grapes, could inhibit sodium currents, with quercetin exhibiting the highest half-maximal inhibitory concentration. While it is still unknown whether quercetin inhibits Nav in sensory neurons, it is plausible to assume that quercetin functions as a stronger Nav channel blocker in nociceptive sensory neurons in vivo compared with other polyphenols.

**Figure 1:**
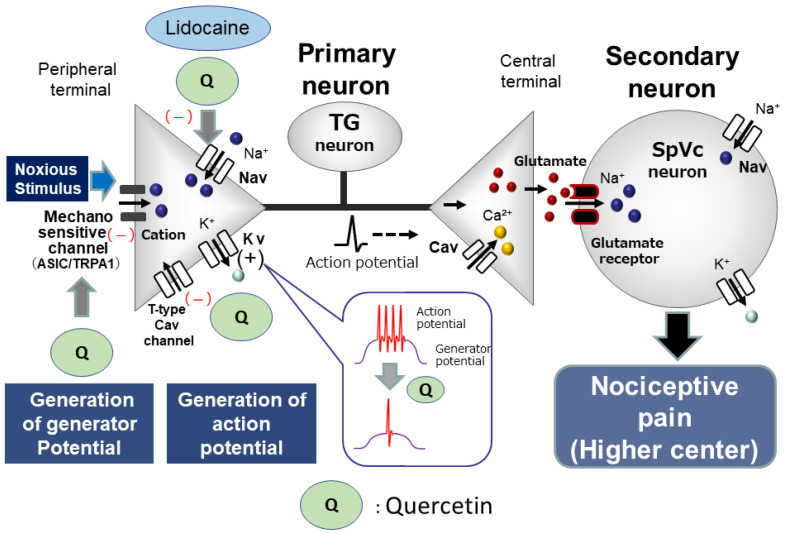
**Possible molecular targets for phytochemicals as local anesthetic agents.** When locally applied to peripheral tissues, quercetin inhibits the development of both generator potentials and action potentials in the peripheral terminal of primary afferents after nociceptive stimulation via the inhibition of mechanosensitive ionic channels (acid-sensing ion channel [ASIC] and transient receptor potential ankyrin 1 [TRPA1]), voltage-gated sodium (Nav) channels, and the facilitation of voltage-gated potassium (Kv) channels. The potency of quercetin is almost equal to that of Nav channel blockers, such as the commonly used local anesthetics, e.g., lidocaine. Cav = voltage-gated calcium, TG = trigeminal ganglion, SpVc = trigeminal spinal nucleus caudalis.

Toyota et al. [19] investigated the impact of the local administration of quercetin in rats to determine if it reduces the excitability of nociceptive TG neurons in vivo in response to mechanical stimulation. They found that *(i)* the mean firing rate of TG neurons in response to both non-noxious and noxious mechanical stimuli is dose dependently reduced by a local injection of quercetin; *(ii)* quercetin inhibition of the discharge frequency in response to both non-noxious and noxious mechanical stimuli is reversible; and *(iii)* a local injection of a vehicle has no significant effect on non-noxious or noxious mechanical stimulation-evoked TG neuronal activity. These findings are in agreement with previous in vitro studies, in which 0.1 mM quercetin was found to inhibit Nav currents in rat cardiac myocytes [13]. Surprisingly, the mean magnitude of TG neuronal discharge frequency inhibition is almost equal between quercetin (10 mM) and 2% lidocaine (74 mM), indicating that the potency of quercetin is seven folds higher than 2% lidocaine. These findings are supported by evidence in rat cardiac myocytes, using a whole-cell patch clamp technique, in which the red grape polyphenols, quercetin, catechin, and resveratrol, all inhibit Nav currents, and their inhibitory potency, based on the half-maximal inhibitory concentration, is quercetin > catechin = resveratrol [13]. These findings indicate that injecting quercetin locally at the peripheral receptive site can inhibit the excitability of nociceptive primary sensory neurons in the TG, likely by blocking Nav channels and activating Kv channels (Figure 1). Since quercetin inhibits ASIC currents in the central vestibular neurons [17], it can be assumed that the local administration of quercetin works through ASICs as candidates for mechanoreceptors in the nerve terminals of TG neurons [39,40]. Therefore, the local administration of quercetin directly inhibits generator potentials, and, subsequently, suppresses the action potential firings of nociceptive TG neurons (Figure 1). The potency of quercetin is almost equal to that of the Nav channel blockers, such as the commonly used local anesthetic, lidocaine.

Alternatively, T-type Cav channels are predominantly expressed in small- and medium-diameter sensory neurons [41,42]. These neurons predominantly consist of unmyelinated C-fibers and myelinated Aδ-fibers. Todorovic and Todorovic [43] observed that an increase in the amplitude of T-type Ca^2+^ currents resulted in a decreased excitability threshold, thereby raising the chance of neuron-firing bursts. A recent study found that quercetin-related compounds, like the flavonoid, gossypetin, exhibited robust analgesic effects in peripheral afferents by interacting with an intracellular signaling pathway that influences T-type Cav channels in dorsal root ganglion (DRG) neurons [38]. Taken together, it can be assumed that quercetin would inhibit the excitability of trigeminal neuronal firing responding to noxious mechanical stimulation. This assumption is supported by a recent report that T-type Cav channels regulate action potential firing and are expressed in TG neurons [44] (Figure 1).

## 6. Local Anesthetic Effect of Quercetin on Acute Inflammatory Pain

Previous studies have demonstrated that ASICs may function as mammalian cutaneous mechanoreceptors in the nerve endings of primary sensory neurons, alongside TRPA1 [8,31,34,35]. Moreover, the application of quercetin inhibits ASIC currents [45]. Under inflamed conditions, tissue pH falls below 6.0, causing the activation of primary nociceptive afferents through ASICs [46]. Based on clinical evidence, local anesthetic efficacy, particularly with dental anesthetics, is diminished by the presence of inflammation [46,47]. Fu et al. [47] discovered that peripheral inflammation has the ability to stimulate the upregulation of ASICs in TG neurons, suggesting that selective ASIC inhibitors may offer substantial relief from orofacial inflammatory pain. All of these results indicate that the direct application of quercetin locally suppresses generator potentials, leading to the inhibition of action potential firing in nociceptive TG neurons by blocking ASIC channels and Nav channels and activating Kv channels in inflamed tissues.

**Figure 2:**
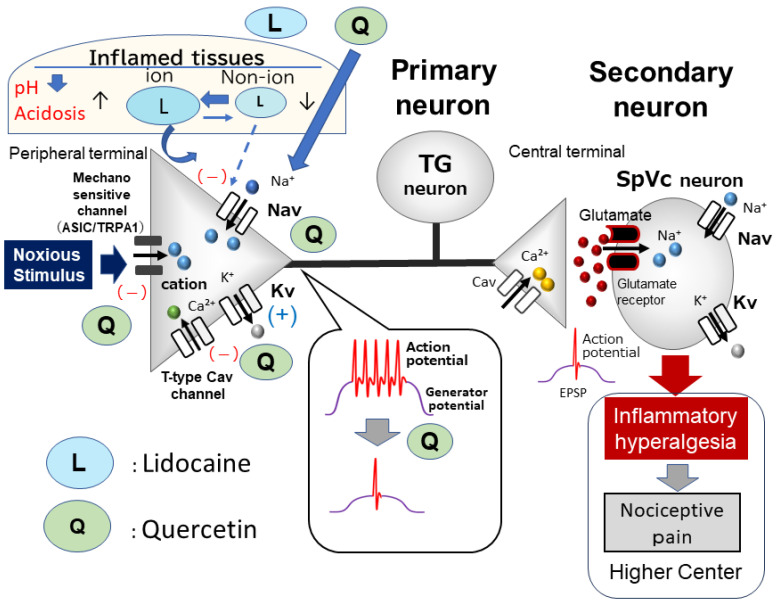
**Local anesthetic effect of quercetin under inflammatory conditions.** The inhibitory effect of quercetin on inflamed tissues may be caused by the suppression of the firing frequency of action potentials by the inhibition of nociceptive mechanosensitive channels (ASIC and TRPA1), the inhibition of Nav channels, the inhibition of T-type Cav channels, and the opening of Kv channels. The inhibitory potency of quercetin on the discharge frequency is significantly higher than that of lidocaine; therefore, quercetin has a strong local anesthetic effect on inflamed tissues and is expected to be used in the field of complementary and alternative medicine. EPSP = excitatory postsynaptic potential.

Recently, Sashide et al. [20] reported that a local injection of quercetin into the peripheral receptive field of rats suppressed the excitability of nociceptive primary sensory neurons in TG neurons as follows. *(i)* The mean firing frequency of TG neurons in response to both non-noxious and noxious mechanical stimuli was reversibly inhibited by quercetin in a dose-dependent manner; *(ii)* the mean firing frequency of inflamed TG neurons in response to mechanical stimuli was reversibly inhibited by the local anesthetic, 1% lidocaine (37 mM); and *(iii)* the mean magnitude of inhibition of TG neuronal discharge frequency with 1 mM quercetin was significantly greater than that of 1% lidocaine [20]. Figure 2 shows that the local application of quercetin in inflamed tissue appears to decrease the excitability of nociceptive primary sensory TG neurons by inhibiting ASICs and Nav and Cav channels while stimulating Kv channels. Therefore, under inflamed conditions, the local administration of the phytochemical, quercetin, may be a more potent local analgesic than the Nav channel blocker, lidocaine, as it inhibits the generation of both generator potentials and action potentials in nociceptive primary nerve terminals [20]. Therefore, quercetin plays a role in the field of CAM. However, further confirmational studies, such as in vitro patch clamp studies of dissociated fluorescently labeled TG neurons derived from the receptive field of inflammatory tissues, are needed [24,48].

Gadotti et al. [38] found that the flavonoid, gossypetin, a molecule with a structure closely resembling quercetin, partially blocks inflammatory and neuropathic peripheral pain conditions by affecting Cav3.2 channels. T-type Cav channels were highlighted by Gambeta et al. [44,49] as essential regulators of neuronal function in the trigeminal system, with a significant impact on trigeminal pain, including trigeminal neuralgia. Ali et al. [37] recently showed that a group of flavonols, such as quercetin, can reduce inflammatory and neuropathic pain by targeting T-type Cav channels through a dual mechanism involving the direct inhibition and modulation of deubiquitination. Notably, quercetin was found to significantly disrupt the interaction between USP5 (deubiquitinase) and Cav3.2. Thus, when considered collectively, these results indicate that quercetin may suppress the responsiveness of trigeminal nerve firing to painful mechanical stimuli by blocking T-type Cav channels in inflammatory situations. However, additional in vitro research is necessary to investigate this possibility.

## 7. Relief from Chronic Inflammatory Pain by Quercetin

**Figure 3:**
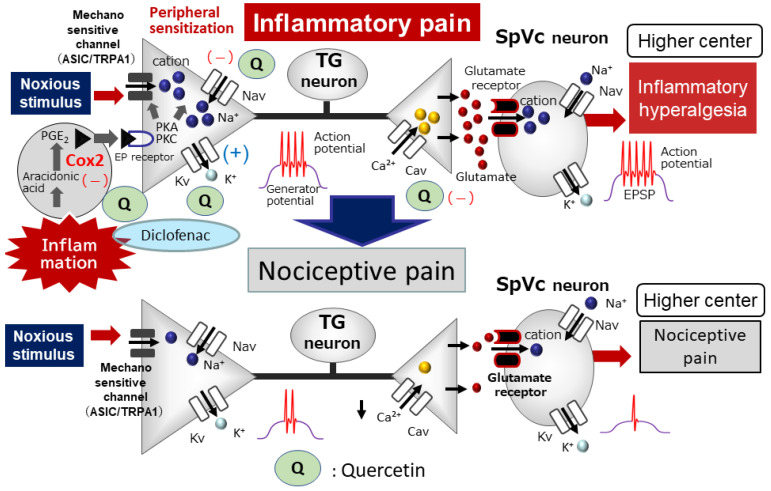
**The systemic administration of quercetin inhibits inflammatory pain**. Following peripheral inflammation, inflammatory mediators, such as prostaglandin E2 (PGE_2_), bind to G-protein-coupled E-type prostanoid (EP) receptors and induce the activation of protein kinase (PK) A and PKC in nociceptive peripheral terminals, leading to the phosphorylation of mechanosensitive (TRPA1, ASIC), Nav, and Kv channels. As a result, the activation threshold for transducer channels, such as the TRP channel family, is reduced, and the membrane excitability of the peripheral terminal increases, resulting in a high frequency of action potentials being conducted to presynaptic central terminals of the SpVc. This results in the release of a large amount of glutamate into the synaptic cleft, which binds to upregulated postsynaptic glutamate receptors, augmenting EPSPs, causing a barrage of action potentials to be conducted to higher centers of pain pathways, and creating a state of heightened sensitivity, termed peripheral sensitization. The systemic administration of quercetin attenuates the mechanical inflammatory hyperalgesia associated with the hyperexcitability of SpVc neurons via the inhibition of peripheral cyclooxygenase-2 (Cox-2) cascade signaling pathways, and this effect restores the SpVc neuronal hyperactivity to control levels. The systemic administration of quercetin also inhibits ASIC, Nav, and Cav channels and opens Kv channels in the peripheral terminals. The potency of quercetin is almost equal to the commonly used analgesics, non-steroidal anti-inflammatory drugs.

Previous research suggests that quercetin treatment reduces pain-related behavior in a model of neuropathic pain [50,51]. Complete Freund’s adjuvant (CFA)-inflamed models are generally well established for trigeminal chronic pain investigations [28,52,53]. It has been demonstrated that the phytochemical, quercetin, controls the Cox-2 signaling pathway, a pathway responsible for the synthesis of PGE_2_, which plays a crucial role in inflammation [14,16]. As shown in Figure 3, PGE_2_ acts as an inflammatory mediator by binding to G-protein-coupled E-type prostanoid receptors on primary sensory neurons that are found in areas of inflammation. It also activates nociceptive ionic channels and Nav channels by phosphorylation. Consequently, the heightened excitability (peripheral sensitization) is amplified by a rise in the amplitude of generator potentials and a reduction in the threshold for activation. As a result, increased excitability (peripheral sensitization) is enhanced by an increase in the amplitude of generator potentials and a decrease in the Nav channel excitatory threshold, resulting in frequent action potentials being conducted to the central nervous system and reaching presynaptic terminals. High concentrations of excitatory neurotransmitters are then released via exocytosis into the synaptic cleft, binding to glutamate receptors on the postsynaptic membrane, increasing the amplitude of EPSPs, and increasing the excitability of nociceptive secondary sensory neurons (central sensitization), and pain information is transmitted to higher centers to induce hyperalgesia (Figure 3). Recently, we investigated whether the chronic administration of quercetin suppresses the hyperexcitability of nociceptive neurons involved in inflammatory hyperalgesia using a CFA-induced inflammation animal model [18]. We demonstrated that in rats with CFA-induced inflammation, the withdrawal reflex threshold when stimulated mechanically with von Frey hairs is considerably lower than in uninjured rats. Two days after the administration of quercetin, central sensitivity associated with inflammation was observed in rats in the inflammation group, resulting in a decrease in the mechanical stimulus threshold, an index of hyperexcitability of SpVc WDR neurons, and an increase in the frequency of spontaneous and evoked discharges. After the administration of quercetin, all signs of central sensitization returned to normal levels [18].

We also found that the systemic administration of quercetin mitigated the CFA-induced inflammatory hyperalgesia and hyperexcitability of SpVc WDR neurons to the level seen in naïve rats [18]. Remarkably, the ability of quercetin (50 mg/kg, i.p.) to inhibit inflammatory hyperalgesia was as effective as that of the NSAID, diclofenac (50 mg/kg, i.p.). Typically, NSAIDs block not only Cox-2 present at inflammation sites but also Cox-1, which is naturally found in the stomach and kidneys, and thus carries the risk of side effects, such as gastrointestinal and renal disorders. Therefore, our findings suggest that the chronic administration of polyphenols reduces inflammatory hyperalgesia by inhibiting the hyperexcitability of SpVc WDR neurons through the inhibition of the peripheral and Cox-2 signaling cascade pathways in nociceptive neurons. The potency of quercetin is almost equal to that of NSAIDs, commonly used as analgesics. Phytochemicals like quercetin can prevent side effects and be used as alternative treatments to NSAIDs for preventing inflammatory hyperalgesia (Figure 3). This idea is also supported by our previous study [54] showing that the lowered mechanical stimulation threshold in CFA-inflamed rats returned to control levels after 3 days of daily administration of the phytochemical carotinoid, lutein. Treatment with this phytochemical compound also reduced the whisker pad’s inflammation-induced swelling back to normal levels and returned the heightened count of Cox-2-immunoreactive cells in the whisker pads of CFA-inflamed rats to baseline levels. In addition, the mean discharge frequency of SpVc WDR neurons to both non-noxious and noxious mechanical stimuli in CFA-inflamed rats was significantly decreased after phytochemical administration. Taken together, these results suggest that the administration of the phytochemical, quercetin, attenuates inflammatory hyperalgesia associated with the hyperexcitability of nociceptive SpVc WDR neurons via the inhibition of the peripheral Cox-2 signaling cascade [18].

## 8. Functional Significance of Orofacial Pain and Perspectives

Individuals undergoing orthodontic procedures commonly develop orofacial pain, both direct tooth pain and pain referred across other areas [55]. However, despite the impact and frequency of such episodes, the physiological mechanism underlying referred pain is unclear [56]. Pain due to tooth movement might be related to post-procedural conditions [57]. Mechanical pressure-induced production of Cox-2-induced proinflammatory mediators can induce inflammatory orthodontic pain [58]. Indeed, Cox-2 production may substantially increase at local inflammation sites, such as periodontal tissue [59]. An immunohistochemical study using a rat model of experimental tooth movement showed Cox-2 in the SpVc [56] but not in periodontal tissue, which suggests that the local inhibition of Cox-2 is important in preventing and mitigating inflammatory pain associated with orthodontic tooth movement. Recently, we investigated the relieving effect of the phytochemical polyphenol, resveratrol, on ectopic hyperalgesia during orthodontic movement treatment in rats fitted with orthodontic appliances and induced experimental tooth movement [60]. We found that resveratrol alleviates ectopic hyperalgesia by inhibiting the hyperexcitability of pain-transmitting SpVc WDR neurons without inhibiting tooth movement. Along with our observations that the systemic administration of quercetin mitigates the CFA-induced inflammatory hyperalgesia and hyperexcitability of SpVc WDR neurons to the level seen in naïve rats [18], these findings suggest that quercetin might attenuate mechanical hyperalgesia by inhibiting SpVc WDR excitability through the Cox-2 signaling pathway. However, further studies are needed to clarify this.

Alternatively, there is strong evidence that the activation of glial cells in the SpVc and the subsequent production of cytokines are important underlying factors in pathological pain [61]. These responses might extend beyond central glial cells to peripheral satellite glial cells. In addition, because peripheral sensitization of trigeminal nociceptors suggests increased neuron–glia interactions [26], we hypothesize that the dietary constituent, quercetin, may be a potential therapeutic agent targeting neuron–glia interactions to inhibit trigeminal inflammatory pain. 

In the future, building on these studies will yield essential knowledge necessary for the advancement of functional foods that effectively alleviate pain, CAM for disease treatment that does not rely on medications, and the development of pain relievers with minimal side effects. This has the potential to significantly improve medical treatment. While the primary emphasis of our research findings in this article has been on confirming the “discriminatory factors” of pain (lateral system), an examination of how phytochemicals affect the “emotional components” of pain (medial system) is also required. Additionally, the research findings outlined in this article will need thorough examination before they can be applied in the clinical setting.

## 9. Conclusions

Quercetin is a flavonoid that is widely found in fruits and vegetables. Since recent studies have demonstrated that quercetin can modulate neuronal excitability in the nervous system, including nociceptive sensory transmission via mechanoreceptors and voltage-gated ion channels, and inhibit the Cox-2 cascade, quercetin may be a CAM candidate [13,14,15,16,17,18,19,20]. CAM has been receiving more attention in recent years as a viable option when conventional drug treatments in Western medicine prove ineffective. Research exploring the potential for alleviating pain by utilizing food ingredients to circumvent the numerous side effects of pharmaceuticals is crucial for advancing “exceptionally safe therapeutic approaches devoid of medication reliance”. As revealed by the research conducted in our laboratory and discussed in this article, we have confirmed in a living system that the phytochemical, quercetin, demonstrates (i) a local anesthetic effect on nociceptive pain, the potency of quercetin being almost equal to lidocaine, a Nav channel blocker commonly used as a local anesthetic; (ii) a local anesthetic effect on pain related to acute inflammation, with quercetin being a more potent local analgesic than Nav channel blockers; and (iii) an anti-inflammatory effect on chronic pain, the potency of quercetin being almost equal to the commonly used analgesics, NSAIDs [18,19,20]. Therefore, these findings suggest that quercetin contributes to the relief of nociceptive and inflammatory pain and implies its potential clinical application.

## Figures and Tables

**Table 1:** Effect of quercetin on the excitable tissue in vitro and in vivo conditions.

Types of Ion Channels	Tissues (naïve/inflamed)	Effect of Quercetin	References
**in vitro**			
● ASICs	Central vestibular neuron (Naïve)	Inhibition	Mukhopadhyay et al. [17]
● Nav	Cardiac myocyte (Naïve)	Inhibition	Wallace et al. [13]
● Kv	Arterial smooth muscle (Naïve)	Facilitation	Hou et al. [15]
● Cav	Arterial smooth muscle (Naïve)	Inhibition	Hou et al. [15]
	Cultured cells (naïve/inflamed)	Inhibition	Ali et al. [37]
**Neuronal** **Excitability/** **Nociceptive** **Behavior**	**Tissues (naïve/inflamed)**	**Effect of Quercetin**	**References**
**in vivo**			
● Noxious Stimulation induced discharge frequency	TG neuron (Naïve)	Inhibition (quercetin = lidocaine)	Toyota et al. [19]
	TG neuron (Inflamed)	Inhibition (quercetin > lidocaine)	Sashide et al. [20]
● Hyperalgesia (nociceptive reflex)	DRG neuron (Inflamed)	Inhibition	Gadotti et al. [38]
	DRG neuron (Inflamed)	Inhibition	Ali et al. [37]

ASICs = acid-sensing ion channels, Nav = voltage-gated sodium channel, Kv = voltage-gated potassium channel, Cav = voltage-gated calcium channels, TG = Trigeminal ganglion, DRG = dorsal root ganglion, = and >: The potency of inhibition.

## Data Availability

Not applicable.
